# Multiparameter resting-state functional magnetic resonance imaging as an indicator of neuropsychological changes in Binswanger’s disease with mild cognitive impairment

**DOI:** 10.3389/fnagi.2025.1522591

**Published:** 2025-02-10

**Authors:** Haiyi Zhang, Lu Zhang, Juan Lu, Jiajun Yue, Zhengzhen Yuan, Jidan Hu, Qi Yao, Yuting Fu, Guiquan Chen, Jiliang Fang, Jie Zhao

**Affiliations:** ^1^Department of Magnetic Resonance Imaging, The Affiliated Traditional Chinese Medicine Hospital, Southwest Medical University, Luzhou, Sichuan, China; ^2^Department of Acupuncture and Rehabilitation, The Affiliated Traditional Chinese Medicine Hospital, Southwest Medical University, Luzhou, Sichuan, China; ^3^School of Physical Education, Southwest Medical University, Luzhou, Sichuan, China; ^4^Department of Radiology, The Second People’s Hospital of Neijiang, Southwest Medical University, Neijiang, Sichuan, China; ^5^Guang’anmen Hospital, China Academy of Chinese Medical Sciences, Beijing, China

**Keywords:** Binswanger’s disease, mild cognitive impairment, multiparameter, functional magnetic resonance imaging, neuropsychological changes

## Abstract

The underlying neuropathological mechanisms in Binswanger’s disease (BD) with mild cognitive impairment (BD-MCI) remain unclear. The multiparameter functional magnetic resonance imaging (fMRI) including amplitude of low-frequency fluctuations (ALFF), fractional amplitude of low-frequency fluctuations (fALFF), regional homogeneity (ReHo), independent component analysis (ICA), and edge-link analysis was utilized to explore the abnormal brain networks of BD-MCI patients. Compared with the BD without MCI group, this study revealed that the ALFF values in the BD-MCI group were significantly increased in the Temporal_Inf_R, Frontal_Mid_Orb_L, and Hippocampus_L, while decreased in the SupraMarginal_R and Precuneus_R. The fALFF value in the BD-MCI group exhibited a reduction in the Frontal_Med_Orb_L. Additionally, ReHo values in the BD-MCI group increased in the Hippocampus_R but decreased in several areas including Precentral_L, Putamen_L, Postcentral_R, Supp_Motor_Area_R, and SupraMarginal_L. The results of ICA revealed that patients diagnosed with BD-MCI exhibited abnormal connectivity patterns across 12 groups of independent components and 5 distinct groups of brain networks. In one group, the internal connectivity within the brain network exhibited abnormalities. The correlation analysis between ALFF and ReHo values and clinical scales revealed a significant negative correlation between the bilateral hippocampus and Mini-Mental State Examination (MMSE) scores. Conversely, ReHo values for Postcentral_R and SupraMarginal_L were significantly positively correlated with MMSE scores. In summary, the results of our study suggest that patients diagnosed with BD-MCI display atypical activity across several brain regions. The observed changes in these areas encompass a range of functional networks. The reduced coordination among these functional networks may play a role in the deterioration of cognitive functions and decision-making capabilities, potentially serving as a critical mechanism contributing to the early manifestation of cognitive impairments.

## 1 Introduction

Binswanger’s disease (BD) is primarily characterized by chronic and progressive subcortical encephalitis caused by extensive microscopic damage to small vessels and nerve fibers in the white matter (Litak et al., 2020). This condition is frequently associated with a gradual decline in cognitive function, as well as an increased risk of stroke, cognitive impairment, and dementia (Litak et al., 2020). BD is reported to be one of the most common causes of dementia after Alzheimer’s disease (AD), accounting for approximately 15% of all dementia cases, yet few patients receive early diagnosis and effective treatment (O’Brien and Thomas, 2015). In clinical practice, patients often present with discomfort arising from complications, and the majority have already undergone significant cognitive decline or even dementia by the time they seek medical intervention (Kalaria, 2018). The cognitive impairment associated with BD imposes substantial economic and psychological burdens on both patients and their families, thereby contributing to a severe societal burden. The insidious progression of the disease, coupled with the absence of characteristic early symptoms and appropriate screening biomarkers, presents significant challenges for ultra-early clinical diagnosis and timely intervention.

Clinical studies on BD have consistently demonstrated neurological alterations in these patients. Structural magnetic resonance imaging (sMRI) studies have revealed structural abnormalities in the hippocampus and its related outflow regions (Liu et al., 2014). A study employing diffusion tensor imaging (DTI) revealed significant microstructural damage in the white matter of patients with BD who exhibit cognitive impairment (Liu et al., 2019). These findings offer compelling evidence that BD induces neurological alterations. Nevertheless, the neurobiological mechanisms underlying cognitive impairments associated with BD remain inadequately understood.

Resting-state functional magnetic resonance imaging (rs-fMRI) reflects a specific type of spontaneous neuronal activity (Raichle and Snyder, 2007). In recent years, blood oxygen level-dependent (BOLD) has served as the foundation for the widespread application of rs-fMRI in investigating various mental disorders, including attention deficit hyperactivity disorder, schizophrenia, bipolar disorder, and autism spectrum disorder (Zhang H. et al., 2024). On this basis, regional homogeneity (ReHo) is utilized to quantify the synchrony of neuronal activity within brain regions (Liang et al., 2024). Meanwhile, the amplitude of low-frequency fluctuations (ALFF) and the fractional amplitude of low-frequency fluctuations (fALFF) serve as indicators of the intensity of spontaneous neuronal activity in these regions (Wei et al., 2024). Furthermore, functional connectivity (FC) assesses the temporal correlation between distinct anatomical brain regions by analyzing the temporal sequence of their neuronal activations (Holub et al., 2023). Independent component analysis (ICA), a statistical technique based on blind source separation algorithms, is employed to identify brain functional networks (Calhoun et al., 2001). Changes in FC both within and between networks can be systematically analyzed.

Prior research has underscored the significant potential of rs-fMRI in examining cognitive deficits among individuals with BD. Methodologies such as seed or region-based FC and ICA are utilized to explore brain network connectivity in BD patients, thereby elucidating the integrative and comprehensive characteristics of interactions between two or more remote brain regions. For instance, Fu et al. (2020) reported an increase in static functional network connectivity (sFNC) between the default mode network (DMN) and sensory regions in BD patients after analyzing 49 intrinsic connectivity networks using fMRI and DTI. Additionally, research conducted by Tu et al. (2020) revealed diminished ReHo values in the frontal lobe of BD patients, indicating potential attention deficits associated with cognitive impairments in this population. Supporting this perspective, additional studies have indicated changes in neuronal activity within the hippocampus and temporal lobe of individuals diagnosed with BD-MCI (Ni et al., 2016). Despite the significance of early cognitive impairment in BD, there has been a relative paucity of research focusing on the neuroimaging mechanisms underlying this condition. Prior investigations have predominantly overlooked the classification and comparison of cognitive impairment severity, and have exhibited a lack of diverse research parameters, which has hindered a comprehensive understanding of the brain function and network characteristics in BD patients experiencing cognitive deficits. We propose that there are distinct neurological alterations associated with early cognitive impairment. In this study, we performed a comparative analysis between BD-MCI patients and those without such impairments to investigate the potential neurological characteristics. This research endeavors to enhance our understanding of the etiology of BD and to identify imaging biomarkers that may predict the onset of cognitive impairment, thereby illuminating the potential patterns of neuronal activity and alterations in brain network connectivity associated with early cognitive impairment.

## 2 Materials and methods

### 2.1 Participants

The study involved a cohort of 31 patients diagnosed with BD accompanied by mild cognitive impairment (BD-MCI) and a control group comprising 31 patients with BD devoid of cognitive impairment (BD-C) (Supplementary Figure 1). All participants were recruited from the outpatient department of the Affiliated Traditional Chinese Medicine Hospital of Southwest Medical University during the period spanning 2022 to 2024. The diagnostic criteria for BD were established in accordance with the clinical diagnostic guidelines proposed by Tu et al. (2017), as well as the clinical research standards delineated by Fu et al. (2020). Additionally, patients diagnosed with BD also satisfied the criteria for subcortical vascular dementia as outlined and the recent consensus statement on BD (Erkinjuntti, 2002; Rosenberg et al., 2016).

### 2.2 Inclusion and exclusion criteria

The inclusion criteria for this study were as follows: (1) all patients met the diagnostic criteria for cognitive impairment as specified in the Diagnostic and Statistical Manual of Mental Disorders, Fifth Edition (Wakefield, 2016) (DSM-V), (2) the age range of 40–60 years, (3) right-handedness, (4) a history of hypertension for a minimum duration of 3 months, (5) presence of abnormal signals confirmed by routine T1 or T2 fluid-attenuated inversion recovery (FLAIR) MRI, with a Fazekas grade of II (Fazekas et al., 1987), (6) Mini-Mental State Examination (MMSE) scores ranging from 16 to 17 for illiterates, 18 to 20 for individuals with primary school education, and 21 to 23 for those with junior high school education or higher, alongside Montreal Cognitive Assessment (MoCA) scores between 18 and 23, indicative of mild cognitive impairment, (7) absence of a history of all-night wakefulness, excessive alcohol consumption, or intake of stimulating substances such as tea or coffee within the 2 days preceding the MRI examination.

Exclusion criteria include: (1) cognitive impairment that is directly attributable to physical conditions or other non-BD, (2) pregnancy, and (3) presence of contraindications for MRI.

The participants in the BD-C group were relatively comparable to those in the BD-MCI group regarding gender, age, and educational background. The inclusion criteria were: (1) age range of 40–60 years, (2) right-handedness, (3) no history of hypertension, (4) presence of abnormal signals confirmed by routine T1 or T2 FLAIR-MRI, with a Fazekas grade of II (Fazekas et al., 1987), (5) MMSE scores ≥17 points for illiteracy, ≥20 points for primary school, ≥24 points for junior high school and above, and ≥24 points for the MoCA, no cognitive impairment, and (6) absence of a history of all-night wakefulness, excessive alcohol consumption, or intake of stimulating substances such as tea or coffee within the 2 days preceding the MRI examination. All subjects provided written informed consent following the principles outlined in the Declaration of Helsinki. The study received approval from the Ethics Committee of the Affiliated Hospital of Traditional Chinese Medicine at Southwest Medical University (KY2023046).

### 2.3 Measurements

To exclude the potential bias associated with the use of a singular scale for evaluating cognitive status outcomes, we implemented a multifaceted approach that involved the utilization of two distinct assessment tools to evaluate the cognitive abilities of all participants. MCI defines an intermediate stage between normal aging and dementia, serving as a significant risk factor for the development of dementia in numerous studies (Petersen, 2016). Informed by prior research, we selected the MMSE and MoCA as the instruments for assessing the cognitive status of the patients. We took into consideration the variations in literacy levels among the participants, as well as findings from previous studies (Li et al., 2024; Ni et al., 2016). We defined a score range of 16–23 for the MMSE and 18–23 for the MoCA as indicative of MCI (Espinoza et al., 2024; Folstein et al., 1975; Suzuki et al., 2024). Each participant completed both the MMSE and MoCA assessments 30 min before the imaging procedure.

### 2.4 fMRI scanning and data preprocessing

All participants underwent examination using a Siemens Skyra 3.0T MRI scanner (Siemens Magnetom Verio, Siemens Medical Systems, Erlangen, Germany), which was equipped with a 16-channel head-neck combined coil, at the Affiliated Hospital of Traditional Chinese Medicine of Southwest Medical University. Each participant affirmed that they remained awake throughout the scanning procedure. During the resting-state scan, participants were instructed to close their eyes, avoid any deliberate thoughts or movements, and have their heads stabilized with foam pads to minimize head motion. Additionally, earplugs were provided to mitigate ambient noise. Each participant was allotted 5–10 min to acclimate to the scanning environment, ensuring they were in a calm and relaxed state prior to the procedure. BOLD images were obtained utilizing a gradient recalled echo (GRE) sequence. Additionally, we were also obtained anatomical T1-weighted whole-brain magnetization-prepared rapid gradient-echo (MPRAGE) images. Please refer to Supplementary Table 1 for specific scanning parameters. This study is based on the MATLAB R2023b platform and uses RESTplus1.28 software (REST)^1^ for preprocessing. The preprocessing follows the author’s previous article, and the process is the same as that of previous studies (Zhang H. et al., 2024). We excluded the data from the initial 10 time points and preprocessed the fMRI data of the subsequent 190 time points in the following order: slice timing, realignment, normalization, smoothing, detrending, nuisance covariates regression, and filtering.

### 2.5 ALFF and fALFF values processing

This part of the treatment also refers to our previous research (Zhang H. et al., 2024). For ALFF analysis, smoothing is essential during the preprocessing stage. Following data preprocessing, the time series of each voxel within the subject’s brain, after being filtered, is transformed into a spectrum using fast Fourier transform (FFT), from which the power spectrum is derived. We then compute the square root of the power spectrum within the frequency range of 0.01–0.08 Hz and designate this as ALFF. Subsequently, the ALFF value of each voxel is divided by the global average ALFF to obtain the standardized zALFF. Normalization of ALFF is achieved by dividing the amplitude of ALFF by the amplitude of the entire frequency band, resulting in fALFF. After standardization, zfALFF is obtained for further statistical analysis.

### 2.6 ReHo values processing

Regional homogeneity analysis eliminating the need for spatial smoothing during data preprocessing. The similarity of the time series of a given voxel to those of its nearest 26 neighboring voxels is quantified using Kendall’s coefficient of concordance (KCC). Individual ReHo values are generated by computing the KCC for each voxel across the entire brain. To normalize the ReHo map, the KCC of each voxel is divided by the mean whole-brain KCC. For all subjects, the KccReHo value of each voxel is further normalized by dividing it by the average ReHo value within the whole-brain mask, resulting in a standardized KccReHo plot (zKccReHo). After applying Gaussian smoothing with a kernel full-width at half-maximum (FWHM) set to 6 mm × 6 mm × 6 mm, the standardized zKccReHo plot is obtained for subsequent statistical analysis.

### 2.7 Independent component analysis

We used the GIFT version 4.0b toolkit^2^ to analyze the preprocessed data, estimating the optimal number of components using the Minimum Description Length (MDL) criterion. In this step, 38 components are selected. Principal component analysis (PCA) was used for dimension reduction at the subject level, and then the data from all subjects were concatenated for a second-dimension reduction. The Infomax algorithm was employed to identify the number of spatially independent components, and the ICASSO algorithm was repeated 20 times to obtain stable independent components. Finally, the group-level ICA was mapped to the individual level, and Fisher-Z transformation was applied to the time series to obtain ICs for each subject. Based on the group-level ICA results, brain network matching was performed using a resting-state template to select the most matching template and independent components with LF/HF > 1.

By integrating both manual matching and automated matching using the GIFT software, we identified a total of 15 independent components within the brain network. To compare the two groups of data, we utilized the Mancovan module in the GIFT toolkit to conduct a two-sample *t*-test, while incorporating age, years of education, and gender as covariates. The validated brain network was then subjected to functional network connectivity (FNC) analysis. Finally, after applying false discovery rate (FDR) correction with a threshold of *P* < 0.05, we obtained statistically significant results.

### 2.8 Edge-based analysis of ROI-wise FC

In this section of the study, we identified all peak brain regions as regions of interest (ROIs) and extracted the mean time series from all voxels within each ROI. We then utilized the RESTplus toolkit to compute the Pearson correlation coefficient between each pair of ROIs. The brain regions were delineated using the AAL template. Subsequently, Fisher’s z transformation was applied to normalize the data and convert it to a corrected distribution. Using the GRETNA toolkit,^3^ we conducted NBS analysis with a two-sample *t*-test, iterating 1,000 times. The effects of age, years of education, and gender were controlled for as covariates.

### 2.9 Analyses of WMHs

Focal white matter hyperintensities (WMHs) were segmented using the FreeSurfer WMH-SynthSeg tool.^4^ The bilateral distance method was employed to determine the distance from each WMH voxel to the ventricles and cortex. Voxels closer to the ventricles were classified as periventricular white matter hyperintensities (PVWMH), while those farther from the ventricles were classified as deep white matter hyperintensities (DWMH).

### 2.10 Statistical analysis

IBM SPSS 26.0 (IBM, Chicago, IL, United States) was used to analyze the demographic and clinical data of the two groups. For the age and education duration data, which followed a normal distribution, an independent samples *t*-test was used. For the remaining indicators, including scores on the MMSE and MoCA scales, the Mann–Whitney *U* test was used. The preprocessed fMRI data were then analyzed using SPM12.0 software (Statistical Parametric Mapping 12)^5^ for a two-sample *t*-test. The cluster level family-wise error (cluster level-FWE) method was chosen for multiple comparison correction, and regions with a corrected *P*-value < 0.05 were considered statistically significant. The Montreal Neurological Institute (MNI) coordinate system was used to locate the peak points of statistically significant regions, which were then named statistically significant brain areas. For the ICA data, we selected a two-sample *t*-test and subsequently applied multiple comparisons correction using the FDR method to the results.

## 3 Results

### 3.1 Clinical data analysis

The results showed that there were no statistically significant differences in gender, age, and education years between the BD-MCI group and the BD-C group (*P* > 0.05). However, the MMSE and MoCA scores of the BD-MCI group were significantly lower than those of the control group, and the differences were statistically significant (*P* < 0.001) (Table 1 and Supplementary Figure 2).

**TABLE 1:** General clinical characteristics of BD-MCI and BD-C.

	BD-MCI group	BD control group(BD-C)	*P*-value
Age, year^a^	53.48 ± 5.94	53.35 ± 7.26	>0.05
Education, year^a^	10.32 ± 5.21	10.42 ± 5.85	>0.05
Gender, *n* (M/F)	31 (15/16)	31 (17/14)	>0.05
MMSE^b^	19.94 ± 2.03	28.06 ± 1.65	<0.001
MoCA^b^	20.39 ± 1.76	28.42 ± 1.67	<0.001

Unless otherwise specified, data are presented as mean ± SD. MMSE, Mini-Mental State Examination; MoCA, Montreal Cognitive Assessment.

*^a^*Two-sample *t*-test.

*^b^*Mann–Whitney *U* test.

### 3.2 Altered ALFF in BD-MCI patients

**FIGURE 1:**
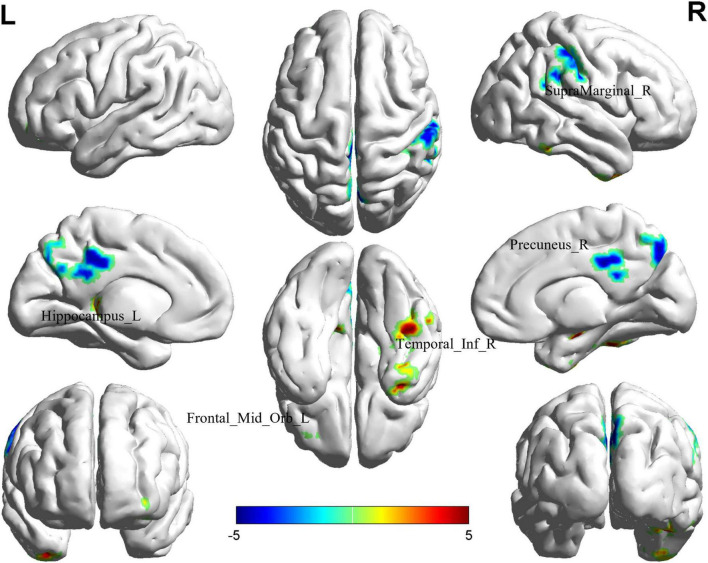
Brain regions (Hippocampus_L, Frontal_Mid_Orb_L, SupraMarginal_R, Precuneus_R, and Temporal_Inf_R) showing significant ALFF in the BD-MCI group compared with the BD-C group on the 3D template (*P* < 0.05, cluster level-FWE). The color scale represents the *T*-value. Cool colors (blue) indicate a significant decrease in value, and warm colors (red) indicate a significant increase in value. R, right; L, left.

Comparison between the BD-MCI group and the BD-C group revealed significant differences in ALFF values in five brain regions (*P* < 0.05, cluster level-FWE corrected; Table 2). The ALFF values were increased in the Temporal_Inf_R, Frontal_Mid_Orb_L, and Hippocampus_L regions (BD-MCI > BD-C; Figure 1 and Supplementary Figure 3), while ALFF values were decreased in the SupraMarginal_R and Precuneus_R regions (BD-MCI < BD-C; Figure 1 and Supplementary Figure 3).

**TABLE 2:** Brain region differences between BD-MCI and BD-C based on ALFF.

Brain regions^a^	Voxel (mm^3^)	AAL	MNI coordinates			*T*-value
			***x***	***y***	***z***	
**BD-MCI > BD-C**						
Cluster 1	357					
Temporal_Inf_R	95	90	45	−18	−21	6.34
Cluster 2	46					
Frontal_Mid_Orb_L	29	9	−30	42	−12	4.18
Cluster 3	45					
Hippocampus_L	24	37	−12	−39	9	4.88
**BD-MCI < BD-C**						
Cluster 4	193					
SupraMarginal_R	130	64	54	−39	27	5.53
Cluster 5	248					
Precuneus_R	56	68	6	−72	45	4.87

*^a^*Peak point of selected brain region cluster.

### 3.3 Altered fALFF in BD-MCI patients

**FIGURE 2:**
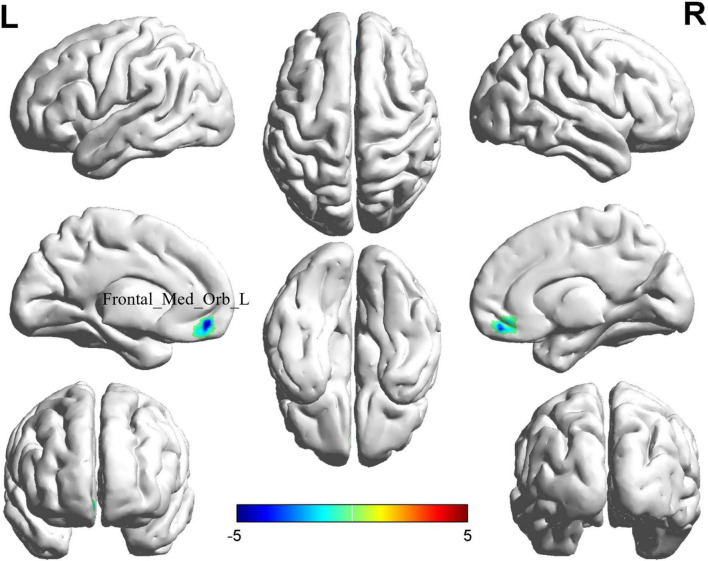
Brain regions (Frontal_Med_Orb_L) showing significant fALFF in the BD-MCI group compared with the BD-C group on the 3D template (*P* < 0.05, cluster level-FWE). The color scale represents the *T*-value. Cool colors (blue) indicate a significant decrease in value, and warm colors (red) indicate a significant increase in value. R, right; L, left.

The comparison between the BD-MCI group and the BD-C group showed that there was a difference in one brain region between the two groups (*P* **<** 0.05, cluster level-FWE corrected; Table 3). The fALFF value was decreased in the Frontal_Med_Orb_L (BD-MCI < BD-C; Figure 2 and Supplementary Figure 4).

**TABLE 3:** Brain region differences between BD and HC based on fALFF.

Brain regions^a^	Voxel (mm^3^)	AAL	MNI coordinates			*T*-value
			***x***	***y***	***z***	
**BD-MCI < BD-C**						
Cluster 1	33					
Frontal_Med_Orb_L	10	25	0	48	−12	4.82

*^a^*Peak point of selected brain region cluster.

### 3.4 Altered ReHo in BD-MCI patients

**FIGURE 3:**
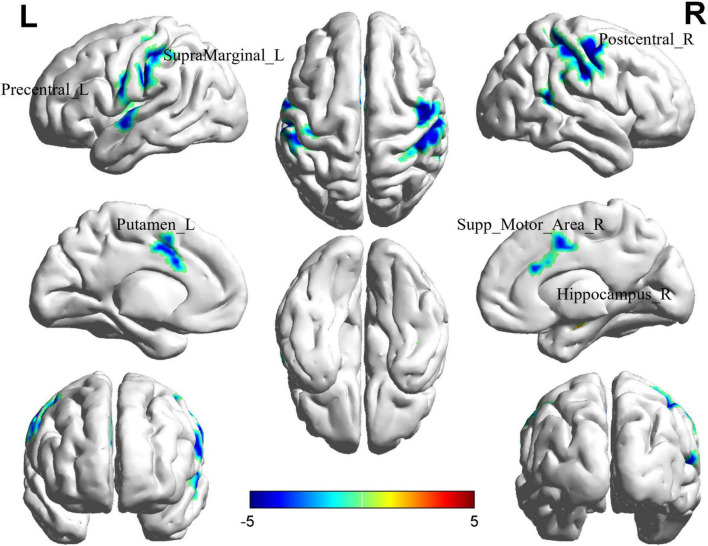
Brain regions (Hippocampus_R, Precentral_L, Putamen_L, Postcentral_R, Supp_Motor_Area_R, and SupraMarginal_L) showing significant ReHo in the BD-MCI group compared with the BD-C group on the 3D template (*P* < 0.05, cluster level-FWE). The color scale represents the *T*-value. Cool colors (blue) indicate a significant decrease in value, and warm colors (red) indicate a significant increase in value. R, right; L, left.

Comparison between the BD-MCI group and the BD-C group revealed significant differences in six brain regions (*P* < 0.05, cluster level-FWE corrected; Table 4). The increased ReHo value was observed in the Hippocampus_R (BD-MCI > BD-C; Figure 3 and Supplementary Figure 5), while decreased ReHo values were observed in the Precentral_L, Putamen_L, Postcentral_R, Supp_Motor_Area_R, and SupraMarginal_L (BD-MCI < BD-C; Figure 3 and Supplementary Figure 5).

**TABLE 4:** Brain region differences between BD-MCI and BD-C based on ReHo.

Brain regions^a^	Voxel (mm^3^)	AAL	MNI coordinates			*T*-value
			***x***	***y***	***z***	
**BD-MCI > BD-C**						
Cluster 1	113					
Hippocampus_R	59	38	39	−18	−15	5.52
**BD-MCI < BD-C**						
Cluster 2	111					
Precentral_L	50	1	−54	−6	27	5.36
Cluster 3	138					
Putamen_L	101	73	−24	6	0	5.70
Cluster 4	527					
Postcentral_R	204	58	54	−15	39	5.24
Cluster 5	99					
Supp_Motor_Area_R	13	20	3	6	48	4.28
Cluster 6	186					
SupraMarginal_L	33	63	−57	−21	33	4.59

*^a^*Peak point of selected brain region cluster.

### 3.5 Alterations of ROI-wise FC in BD-MCI patients

**FIGURE 4:**
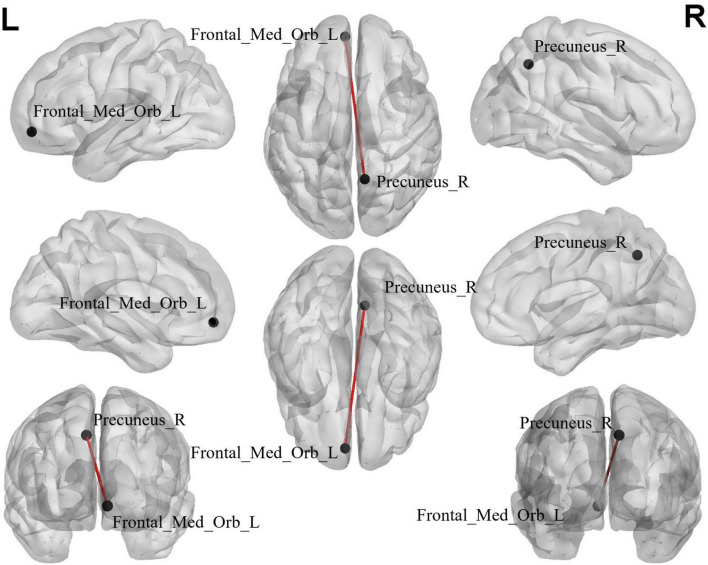
The black dots represent brain ROI, and the red lines represent brain regions that show a decline in functional brain connectivity (*P* < 0.000999001, NBS-corrected). R, right; L, left.

The edge-based analysis of ROI-wise FC comparisons revealed that there was a significant difference in only one group of brain regions between the two groups (*P* < 0.000999001, NBS-corrected; Table 5). The functional connection between the Precuneus_R and Frontal_Med_Orb_L brain regions decreased (Figure 4).

**TABLE 5:** Differences in edge-based analysis based on ROI-wise FC (seed-seed).

ROI (seed)	Brain regions (seed)	*P-*value
Precuneus_R	Frontal_Med_Orb_L	0.0001

### 3.6 Alterations of the FNC brain network in BD-MCI patients

**FIGURE 5:**
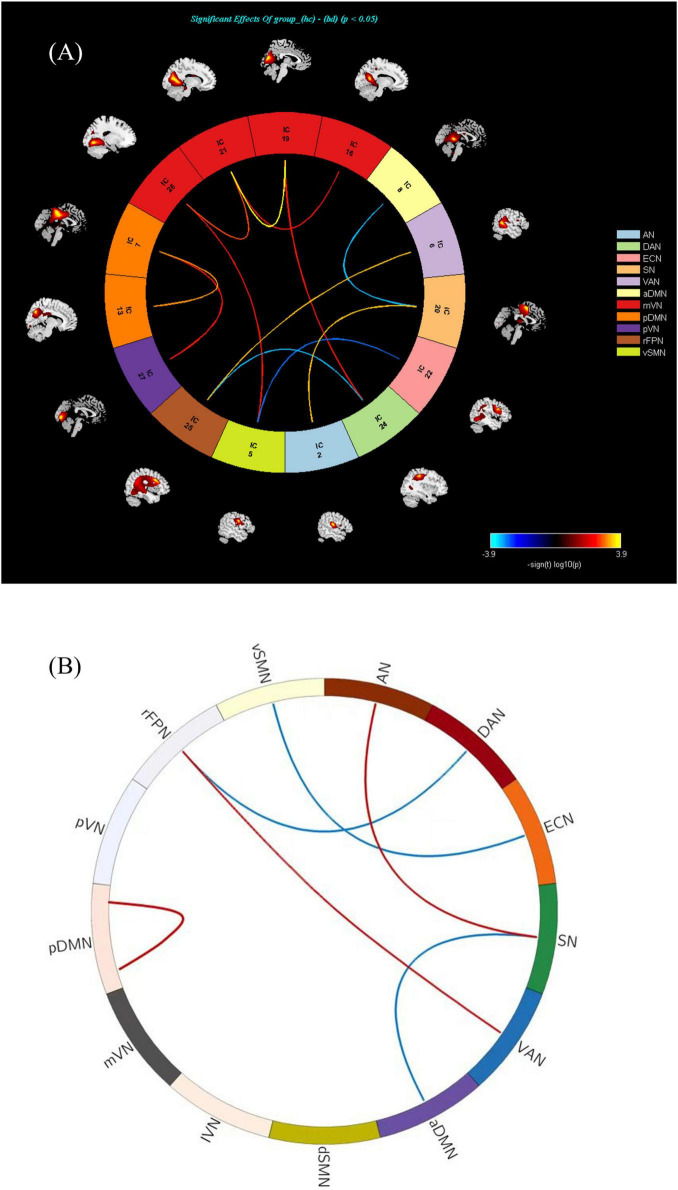
**(A)** There is abnormal connectivity between 12 groups of independent components (*P* < 0.05, FDR corrected). Warmer colors (positive values) represent a strengthened correlation between the two groups, while cooler colors (negative values) represent a weakened correlation. **(B)** There exists an abnormal connectivity among five groups of brain networks (*P* < 0.05, FDR corrected). There was one group of abnormal internal connections identified within a brain network, characterized by enhanced connectivity within the pDMN (*P* < 0.05, FDR corrected). Warmer colors (positive values, red) represent a strengthened correlation between the two groups, while cooler colors (negative values, blue) represent a weakened correlation.

The comparison between the BD-MCI group and the BD-C group showed that a total of 12 groups of independent components had abnormal connections (*P* < 0.05, FDR-corrected; Table 6, Figure 5A, and Supplementary Figures 6, 8). There were five abnormal connections between brain networks, with enhanced connectivity between rFPN-VAN and AN-SN, and weakened connectivity between rFPN-DAN, vSMN-ECN, and aDMN-SN (Table 7, Figure 5B, and Supplementary Figures 7, 9). There was one group of abnormal internal connections within a brain network, with enhanced connectivity within pDMN-pDMN (Table 8, Figure 5B, and Supplementary Figures 7, 9).

**TABLE 6:** Connectivity differences between independent components.

Independent components (ICs)	*T-*value^a^	*P-*value
IC2-IC20	3.7198	0.0005
IC26-IC5	3.0506	0.0034
IC24-IC19	3.0649	0.0033
IC16-IC21	3.1032	0.0029
IC19-IC21	4.1006	0.0001
IC21-IC26	3.5266	0.0019
IC7-IC13	3.5272	0.0008
IC7-IC27	3.0800	0.0031
IC6-IC25	3.7552	0.0005
IC22-IC5	−3.3455	0.0014
IC24-IC25	−3.6540	0.0015
IC20-IC8	−3.5984	0.0007

*^a^*A positive *T*-value indicates increased connectivity, while a negative *T*-value indicates decreased connectivity.

**TABLE 7:** Connectivity differences between brain networks.

Brain network	*T-*value^a^	*P-*value
rFPN-VAN	3.7552	0.0004
AN-SN	3.7198	0.0005
rFPN-DAN	−3.6540	0.0005
vSMN-ECN	−3.3455	0.0015
aDMN-SN	−3.2667	0.0018

*^a^*A positive *T*-value indicates increased connectivity, while a negative *T*-value indicates decreased connectivity.

**TABLE 8:** Alterations in connectivity patterns within brain networks.

Brain network	*T-*value^a^	*P-*value
pDMN-pDMN	3.5272	0.0008

*^a^*A positive *T*-value indicates increased connectivity.

### 3.7 Correlation analysis of ALFF and ReHo value with clinical scales

**FIGURE 6:**
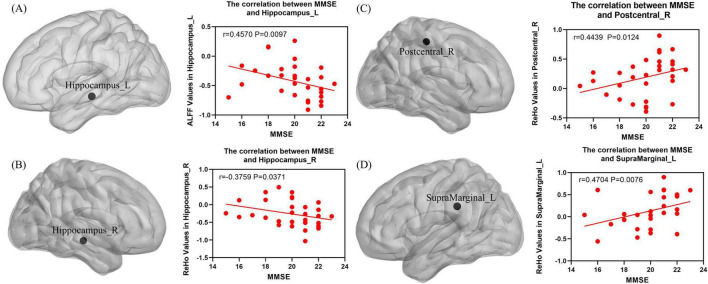
**(A)** The ALFF value of the Hippocampus_L exhibited a significant negative correlation with the MMSE score (*P* < 0.05, Spearman correlation analysis). **(B)** The ReHo value of Hippocampus_R exhibited a significant negative correlation with the MMSE score (*P* < 0.05, Spearman correlation analysis). **(C)** The ReHo value of Postcentral_R exhibited a significant positive correlation with the MMSE score (*P* < 0.05, Spearman correlation analysis). **(D)** The ReHo value of SupraMarginal_L exhibited a significant positive correlation with the MMSE score (*P* < 0.05, Spearman correlation analysis).

The correlation analysis of ALFF and ReHo with clinical scales showed that the ALFF value of Hippocampus_L was significantly negatively correlated with the MMSE score (*P* < 0.05, Spearman correlation analysis; Figure 6A), the ReHo value of Hippocampus_R was significantly negatively correlated with the MMSE score (*P* < 0.05, Spearman correlation analysis; Figure 6B), and the ReHo values of Postcentral_R and SupraMarginal_L were significantly positively correlated with the MMSE score (*P* < 0.05, Spearman correlation analysis; Figures 6C, D).

### 3.8 WMHs analysis results

The analysis of WMHs revealed that the BD-MCI group exhibited a significantly greater volume of DWMH (*P* = 0.0086; Supplementary Figure 10). Additionally, there was a negative correlation between the volume of DWMH and MMSE scores (*P* < 0.05, Spearman correlation analysis; Supplementary Figure 11).

## 4 Discussion

In this study, the BD-MCI patient group demonstrated significant alterations in ALFF, fALFF, ReHo, and ROI-wise FC across various brain regions, including the frontal, parietal, and temporal lobes. ICA indicates significant abnormalities in the connections between multiple brain networks and within brain networks in the BD-MCI patient group. Increased ALFF, fALFF, and ReHo values may indicate a high level of neuronal activation and abnormal neural activity in specific brain region, while decreased measurement values represent a reduction in overall neural activity in the same region. The modulation of connections within the brain network, whether enhanced or diminished, indicates alterations in the collaborative capabilities of both intra- and inter-network among this cohort of patients. These results highlight the fact that people with BD-MCI exhibit abnormalities in neuronal activity, coherence, and network connectivity throughout many brain regions. The cognitive deficits observed in BD patients may be linked to these irregularities affecting both regional function and network collaboration.

Previous studies have unequivocally established that the frontal cortex is integral to higher-order cognitive functions, vigilance, and attention (Li et al., 2016). Research conducted by Zhao et al. (2023) on brain structure in patients with mild cognitive impairment demonstrated that such impairments are associated with a reduction in gray matter volume within the frontal cortex. This abnormality suggests potential clinical manifestations, including deficits in cognition, behavior, psychological wellbeing, and emotional expression (Zhao et al., 2023). Furthermore, Zhao et al. (2023) observed that the fALFF value in the medial superior frontal gyrus of patients suffering from cognitive impairment due to coronary heart disease was significantly lower than that of healthy controls, corroborating the findings of this study. Additionally, another investigation from Yue et al. (2023)

revealed that both the ALFF value and ReHo value of the left superior frontal gyrus were increased in patients with amnestic MCI compared to healthy individuals. These values exhibited a significant negative correlation with scores on the MoCA Scale. In our study, we identified an increase in ALFF values within the left middle frontal gyrus. We hypothesize that this may reflect compensatory neural activity within the prefrontal cortex aimed at offsetting declines in cognitive function. This aligns with prior research suggesting that individuals with amnestic MCI may engage additional neural resources within prefrontal regions as compensation for their cognitive deficits (Yue et al., 2023).

The parietal lobe is implicated in numerous high-level cognitive processes such as episodic memory retrieval, self-referential information processing, and various dimensions of consciousness (Marques et al., 2018; Zhang Y. et al., 2024). The precuneus, as a component of the parietal lobe, has been demonstrated to play a pivotal role in various highly integrated tasks (Cavanna and Trimble, 2006). In our research cohort comprising patients exhibiting BD, we found significantly reduced ALFF values within the precuneus. Zhang X. et al. (2024) reported alterations in the fALFF value of the precuneus in AD patients, suggesting that these changes were closely associated with cognitive impairment experienced by the patients. Additionally, a study conducted by Costa et al. (2024) indicated that local connectivity within the precuneus was diminished in individuals with MCI, further underscoring the significance of reduced neural activity in this region during episodes of cognitive decline. Meanwhile, we also observed decrease in the ALFF and ReHo values of the supramarginal gyrus, as well as a reduction in the ReHo value of the postcentral gyrus. Recent studies have demonstrated that abnormal activities in these brain regions are directly associated with cognitive impairments in patients. Hoshi et al. (2023) proposed through their research on large-scale clinical datasets that reduced beta-band activity in the supramarginal gyrus reflects a decline in cognitive abilities. Mu et al. (2024) suggested that the fALFF value of the postcentral gyrus would decrease proportionally with the severity of cognitive impairment. The alterations observed in these brain regions indicate significant dysregulation within the parietal lobe of patients suffering from BD-related cognitive disorders, which is closely correlated with the pathogenesis of such disorders.

Additionally, we hypothesized that BD-MCI patients had broad increases in brain activity in the inferior temporal gyri and hippocampus areas. This finding was consistent with previous conclusions drawn by Zhang et al. (2021). The relationship between hippocampal function and human memory has been extensively validated over time, emphasizing its multifaceted roles in essential cognitive functions such as memory processing, storage, and spatial information management (Kumaran and Maguire, 2005). Studies investigating electrical stimulation applications for treating post-stroke cognitive impairments have demonstrated a reduction in ALFF values for the hippocampi of treated model rats (Wen et al., 2018). In a task-related fMRI study involving patients with mild cognitive impairment, overactivity was noted in the hippocampal region during the memory encoding process (Tao et al., 2019). Consistent with prior research, similar patterns were identified in the inferior temporal gyrus (Pan et al., 2017). This suggests that heightened arousal states within both the hippocampus and inferior temporal gyrus significantly contribute to cognitive deficits. Notably, neuroimaging analysis conducted by Chen et al. (2017) revealed a substantial reduction in gray matter volume and ReHo of the left putamen, which closely aligns with our current findings.

In our study examining the impact of ALFF, fALFF, and ReHo values on neuronal activity in the brain, we observed that certain brain regions exhibited abnormalities across multiple metrics. Specifically, as a critical region associated with cognitive disorders, the ALFF and ReHo values were found to be reduced in the BD-MCI group within this area. This reduction may be attributed to more severe disturbances and diminished neuronal activity in this region. Consistent with prior research by Ren et al. (2024) on AD, we hypothesize that decreased activity in the supramarginal gyrus could be a shared characteristic between BD-MCI and AD patients, potentially indicating an important brain region implicated in cognitive diseases. Similarly, in the hippocampus, ALFF and ReHo values were elevated in the BD-MCI group, suggesting that heightened hippocampal arousal might be an early manifestation of cognitive impairment, aligning with findings by Hao et al. (2019) and Yue et al. (2023) regarding MCI. These regions can serve as focal points for further investigation.

Brain networks perform distinct yet critically important roles in human activities. The connectivity between these brain networks was equally vital for understanding alterations associated with BD-MCI patients. Among these networks, structural and functional abnormalities within the DMN were prominent characteristics linked to the onset and progression of cognitive impairment (Mencarelli et al., 2024). The DMN is a FC network that operates during resting states, automatically collecting, processing, and storing information from external environments. This network encompasses a diverse array of functions, including episodic memory, semantic extraction, and emotional processing (Raichle and Snyder, 2007). Our study identified an abnormal increase in internal connectivity within the DMN. Concurrently, we observed atypical changes in activity levels among regions including the precuneus, medial prefrontal cortex, inferior parietal lobule, and temporal lobe cortex which consist of the DMN. Notably, connectivity between the precuneus and medial prefrontal cortex was diminished. Drawing on prior reports alongside our observations regarding internal functional changes within the DMN, we hypothesized that its aberrant activity may significantly contribute to elucidating potential mechanisms associated with BD-MCI patients (Raichle and Snyder, 2007).

The SN plays a crucial role in processing sensorimotor information as well as overall cognition and emotion regulation (Chang et al., 2013). It facilitates transitions between internal and external processing by modulating two primary control networks in the brain, concluding DMN and ECN. In this study, we observed a decrease in connectivity between SN and DMN, suggesting impaired coordination between these two brain networks during internal cognitive processes (Elton and Gao, 2014; Goulden et al., 2014). Liu et al. (2024) reported similar findings. They noted reduced SN-DMN connectivity in patients with mild traumatic brain injury. This alteration was associated with diminished cognitive abilities among those individuals. Furthermore, our research revealed an enhancement in connectivity between the auditory network (AN) and SN. We hypothesized which may represent a unique manifestation related to relatively early stages of disease progression or could serve as a potential compensatory mechanism aiming at preserving cognitive function. Additional studies were warranted to further explore the alterations within the AN-SN pathway, considering the scarcity of existing studies on this field and the varied viewpoints reflected in the current literature.

The integration of the ECN and SMN was crucial for effectively perceiving and responding to information (Rashidi-Ranjbar et al., 2023). Disorders in connectivity between these networks can result in impaired cognitive function. Research indicated that elderly individuals with weakened ECN-SMN pathway connectivity face a heightened risk of developing cognitive impairment, which aligns with our current study’s findings. Furthermore, we identified declined connectivity within the vSMN-ECN network among patients with BD-MCI, suggesting that interactions between SMN and ECN may significantly contribute to the pathogenesis of cognitive impairment.

Our investigation uncovered abnormalities in the connectivity of the rFPN concerning both the DAN and VAN. The FPN commonly referred to as the cognitive control network, primarily facilitates functions such as working memory and cognitive processing (Cole et al., 2013). The DAN is responsible for sustaining attention, encompassing aspects like alertness, selectivity, and processing capabilities which were elements integral to most cognitive activities (Castiello and Paine, 2002). In our research, we identified weakened connectivity within the rFPN-DAN pathway, indicating compromised attentional and executive control abilities in patients suffering from vascular dementia secondary to BD-MCI. Interestingly, the connectivity within the rFPN-VAN pathway was found to be augmented in this investigation. The VAN was essential for refocusing attention during cognitive activities, this improvement could be explained by aberrant brain connections brought on by BD-MCI patients’ inability to focus. Therefore, maintaining concentration may serve as a crucial strategy for delaying the progression of cognitive impairments.

White matter hyperintensities research has been extensively applied to the study of cognitive impairment-related diseases. Hirao et al. (2022) and Weng et al. (2023) investigated the potential correlation between cognitive disorders and WMH volume. Our study revealed a significant negative correlation between DWMH volume and cognitive function, suggesting that DWMH volume may be a critical factor contributing to cognitive impairment.

This study has several limitations as well. The sample size is relatively small, which may impact the results. Patients with cognitive impairments stemming from other diseases or those with primary cognitive disorders were not included in this analysis. Despite utilizing multiple assessment scales, evaluations were somewhat constrained by a lack of objective measurements. Since the related research on the cerebellum is not fully mature, we did not include the cerebellum region in this study. Thus, we plan to conduct follow-up research involving a larger sample size to compare cognitive impairments across BD and AD alongside primary cognitive disorders. We will also analyze the cerebellar hemispheres individually, looking for specific changes in the cerebellar hemispheres. Additionally, we seek to identify more objective methods for assessing cognitive situations.

## 5 Conclusion

In summary, the results of our study suggested that individuals of BD with MCI display atypical activity across several brain regions. The observed changes in these areas encompass a range of functional networks, including the DMN, SN, ECN, DAN, and VAN. The reduced coordination among these functional networks may play a role in the deterioration of cognitive functions and decision-making capabilities, potentially serving as a critical mechanism contributing to the early manifestation of cognitive impairments.

## Data Availability

The original contributions presented in this study are included in this article/Supplementary material, further inquiries can be directed to the corresponding authors.

## Footnotes

1
http://restfmri.net

2 http://mialab.mrn.org/software/gift/index.html, accessed on 5 June 2022.

3
https://www.nitrc.org/projects/gretna

4
http://surfer.nmr.mgh.harvard.edu/fswiki/WMH-SynthSeg

5
http://www.fil.ion.ucl.ac.uk/spm/software/spm12/
