# Supplementary material for: Multiparameter resting-state functional magnetic resonance imaging as an indicator of neuropsychological changes in Binswanger’s disease with mild cognitive impairment
Source: Front Aging Neurosci. 2025 Feb 10;17:1522591. doi: 10.3389/fnagi.2025.1522591 (PMC11847846; doi:10.3389/fnagi.2025.1522591)
Supplement: Supplementary file 1 [file Data_Sheet_1.pdf]

Supplementary Material

Multiparameter Resting-State Functional Magnetic Resonance Imaging as an Indicator of Neuropsychological Changes in Binswanger' s Disease with Mild Cognitive Impairment

Contents

Table S1: Scan parameters for the BOLD and T1-MPRAGE sequences.

Figure S1. Study flow chart.

Figure S2. General clinical characteristics of BD-MCI and BD-C.

Figure S3. Brain regions (Hippocampus_L, Frontal_Mid_Orb_L, SupraMarginal_R, Precuneus_R, Temporal_Inf_R) showing significant ALFF in the BD-MCI group compared with the BD-C group on the transverse ($P < 0.05$, GRF corrected). The color scale represents the t-value.

Figure S4. Brain regions (Frontal_Med_Orb_L) showing significant fALFF in the BD-MCI group compared with the BD-C group on the 3D template ($P < 0.05$, GRF corrected). The color scale represents the t-value.

Figure S5. Brain regions (Hippocampus_R, Precentral_L, Putamen_L, Postcentral_R, Supp_Motor_Area_R, SupraMarginal_L) showing significant ReHo in the BD-MCI group compared with the BD-C group on the 3D template ($P < 0.05$, GRF corrected). The color scale represents the t-value.

Figure S6. FNC correlations matrix (averaged over subjects).

Figure S7. FNC correlations domain matrix (averaged over subjects).

Figure S8. The effect of experimental group and control group was significant (between independent components).

Figure S9. The effect of experimental group and control group was significant (between brain networks).

Figure S10. The analysis of WMHs revealed that the BD-MCI group exhibited a significantly greater volume of DWMH.

Figure S11. The volume of DWMH was negatively correlated with MMSE score.

Table S1: Scan parameters for the BOLD and T1-MPRAGE sequences.

fMRI sequence parameter	BOLD parameter	T1-MPRAGE parameter
repetition time (TR)	2720ms	1960ms
echo time (TE)	40ms	2.98ms
flip angle	90°	90°
thickness/gap	4.0/0 mm	1.0/0 mm
field of view (FOV)	240 mm×240 mm	256 mm×256 mm
in-plane resolution	64×64	256×256
axial slices, acquisition time points/sagittal slices	270	176
total acquisition time	12 min 20 sec	4 min 42 sec

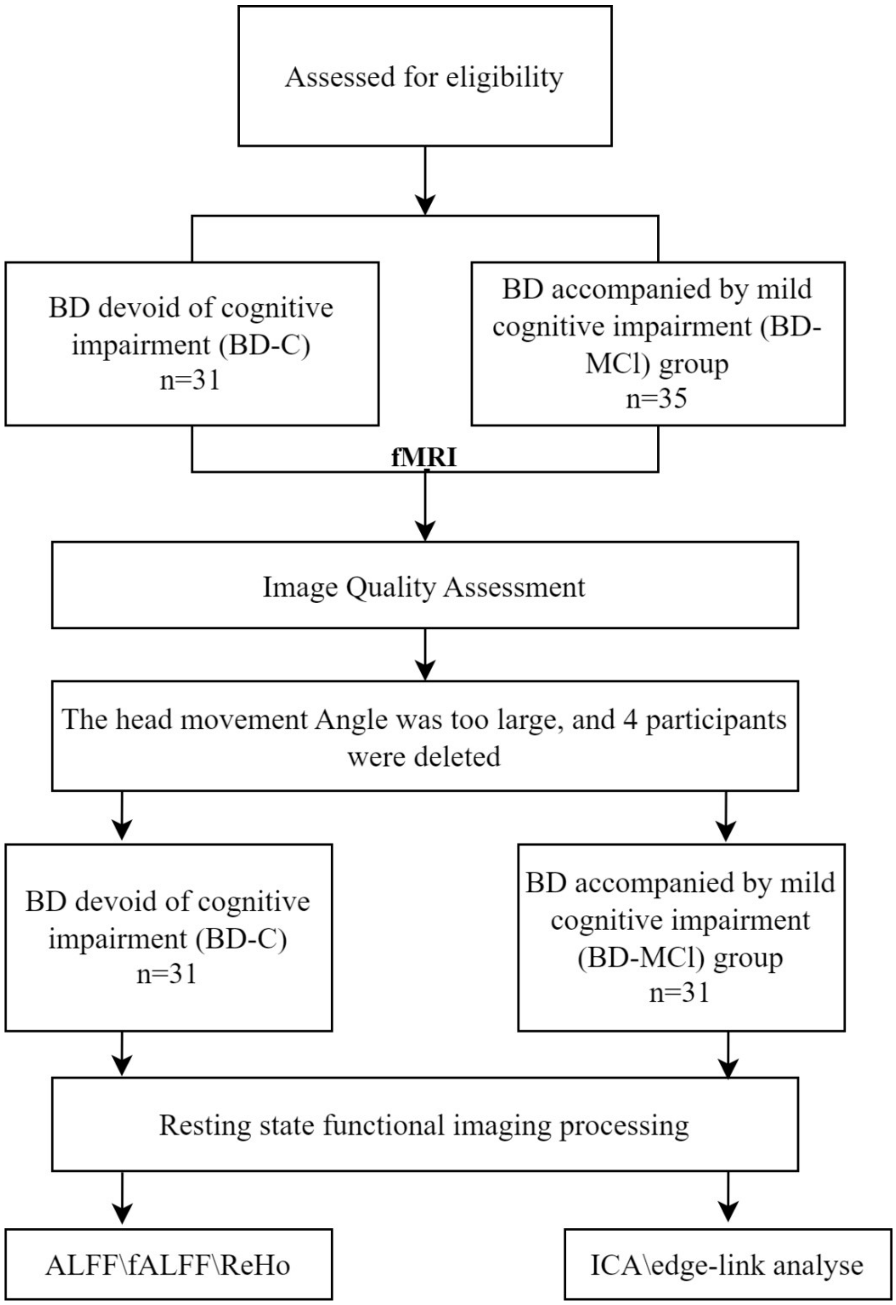

Figure S1. Study flow chart.

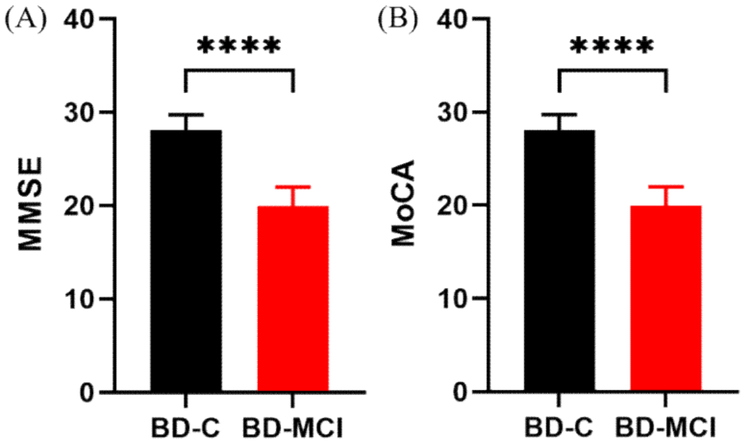

Figure S2. (A) The scores of the MMSE in the BD-MCI group were markedly lower than those observed in the control group (BD-C), with these differences reaching statistical significance ($P < 0.001$). (B) The scores of the MoCA in the BD-MCI group were markedly lower than those observed in the control group (BD-C), with these differences reaching statistical significance ($P < 0.001$).

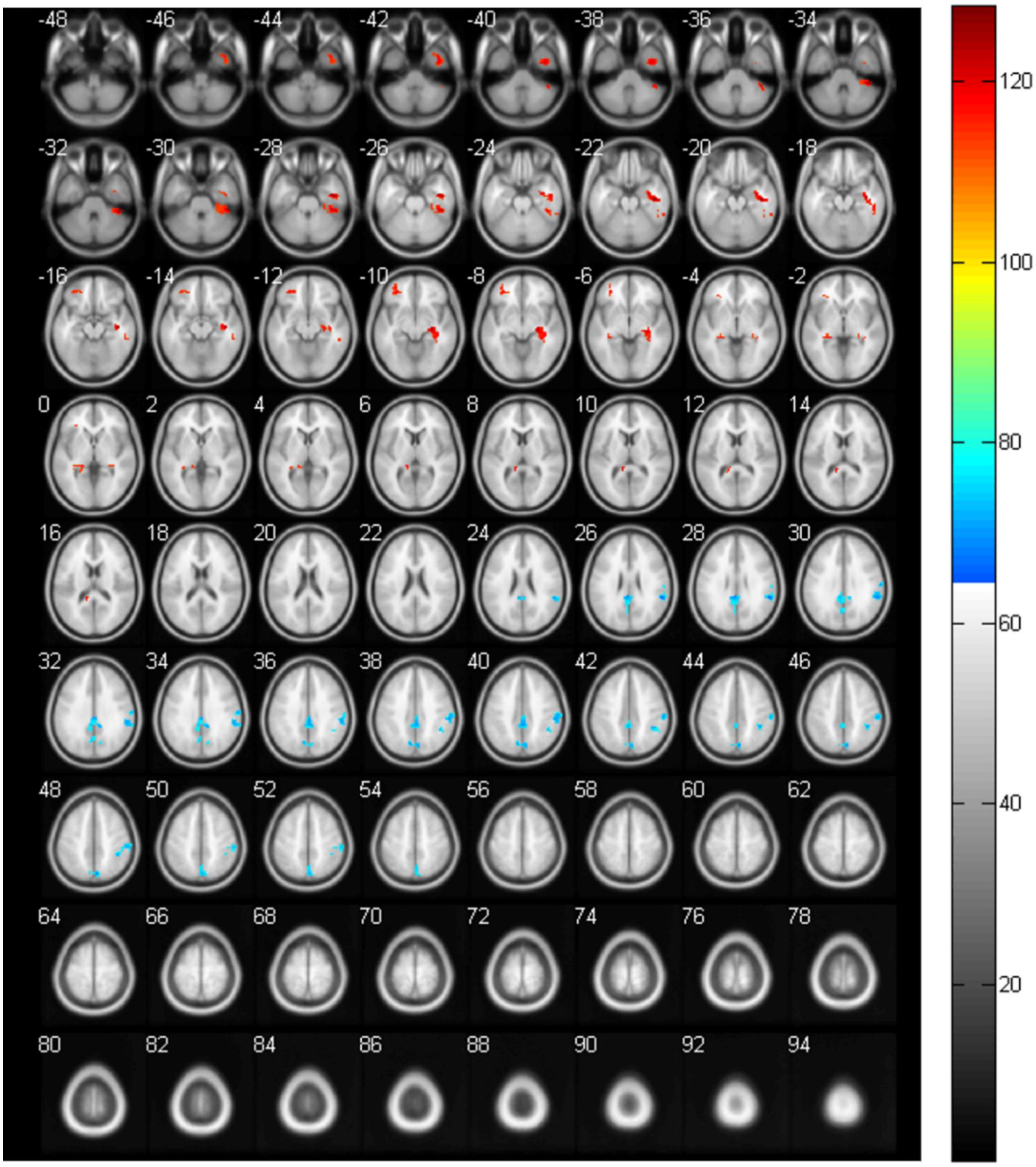

Figure S3. Brain regions (Hippocampus_L, Frontal_Mid_Orb_L, SupraMarginal_R, Precuneus_R, Temporal_Inf_R) showing significant ALFF in the BD-MCI group compared with the BD-C group on the transverse ($P < 0.05$, GRF corrected). The color scale represents the t-value.

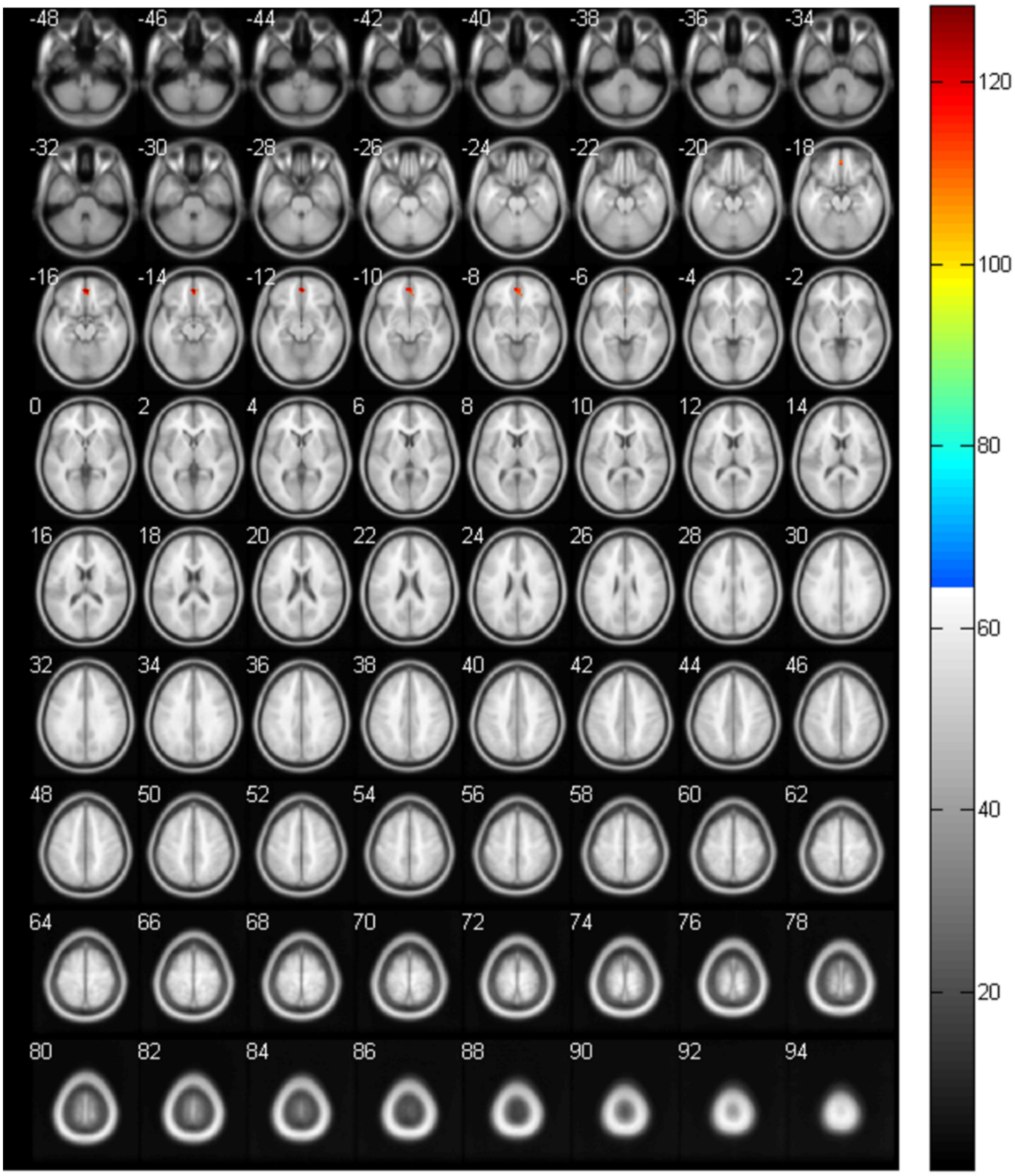

Figure S4. Brain regions (Frontal_Med_Orb_L) showing significant fALFF in the BD-MCI group compared with the BD-C group on the 3D template ($P < 0.05$, GRF corrected). The color scale represents the t-value.

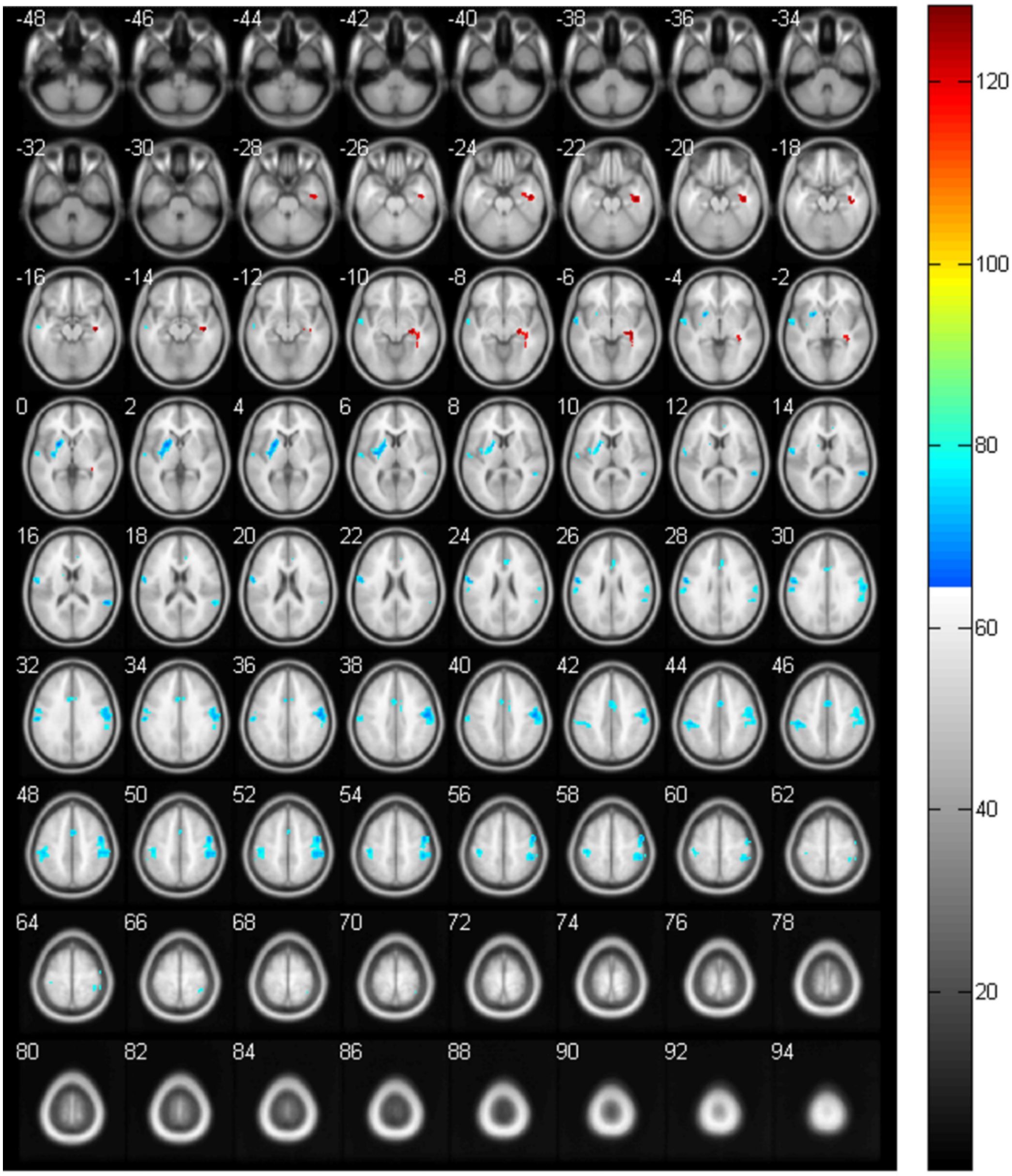

Figure S5. Brain regions (Hippocampus_R, Precentral_L, Putamen_L, Postcentral_R, Supp_Motor_Area_R, SupraMarginal_L) showing significant ReHo in the BD-MCI group compared with the BD-C group on the 3D template ($P < 0.05$, GRF corrected). The color scale represents the t-value.

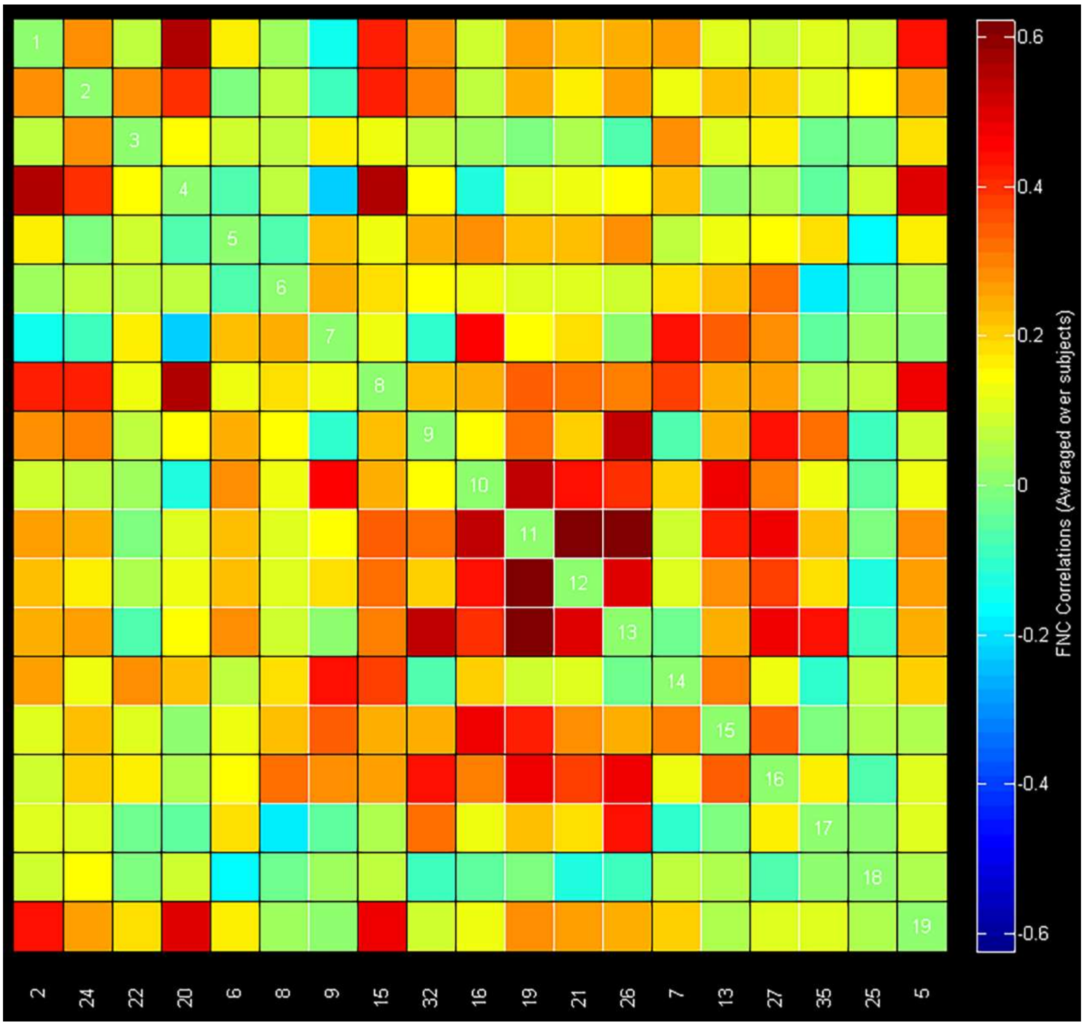

Figure S6. FNC correlations matrix (averaged over subjects).

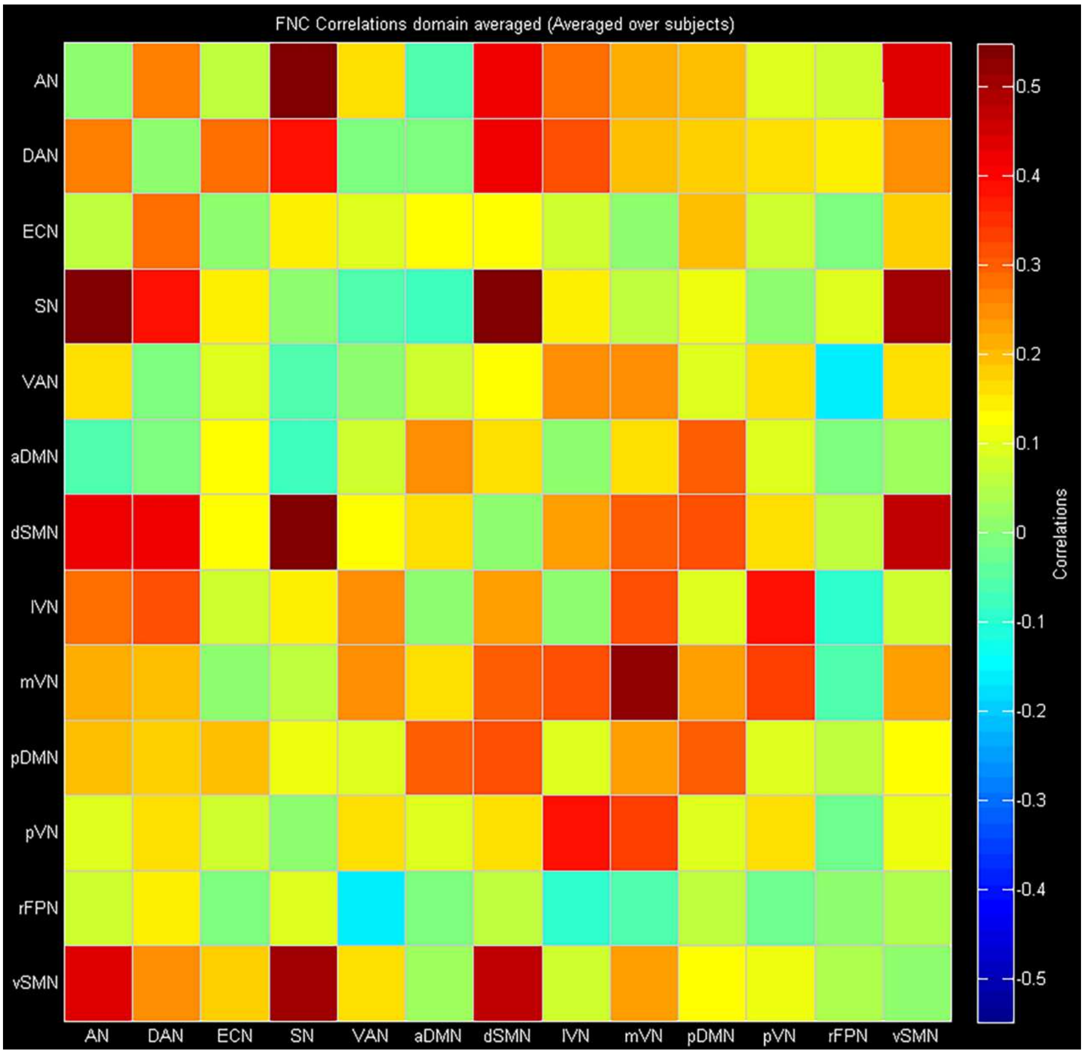

Figure S7. FNC correlations domain matrix (averaged over subjects).

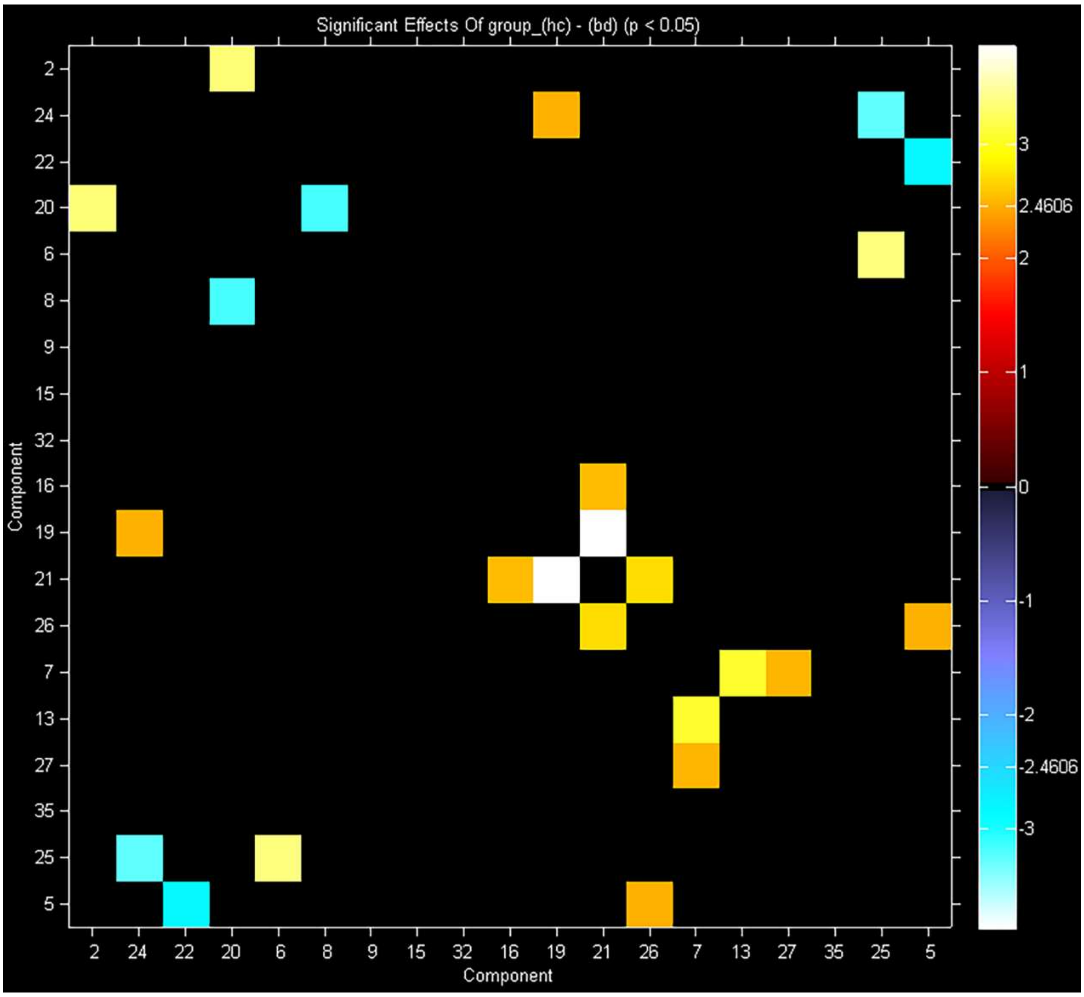

Figure S8. The effect of experimental group and control group was significant (between independent components).

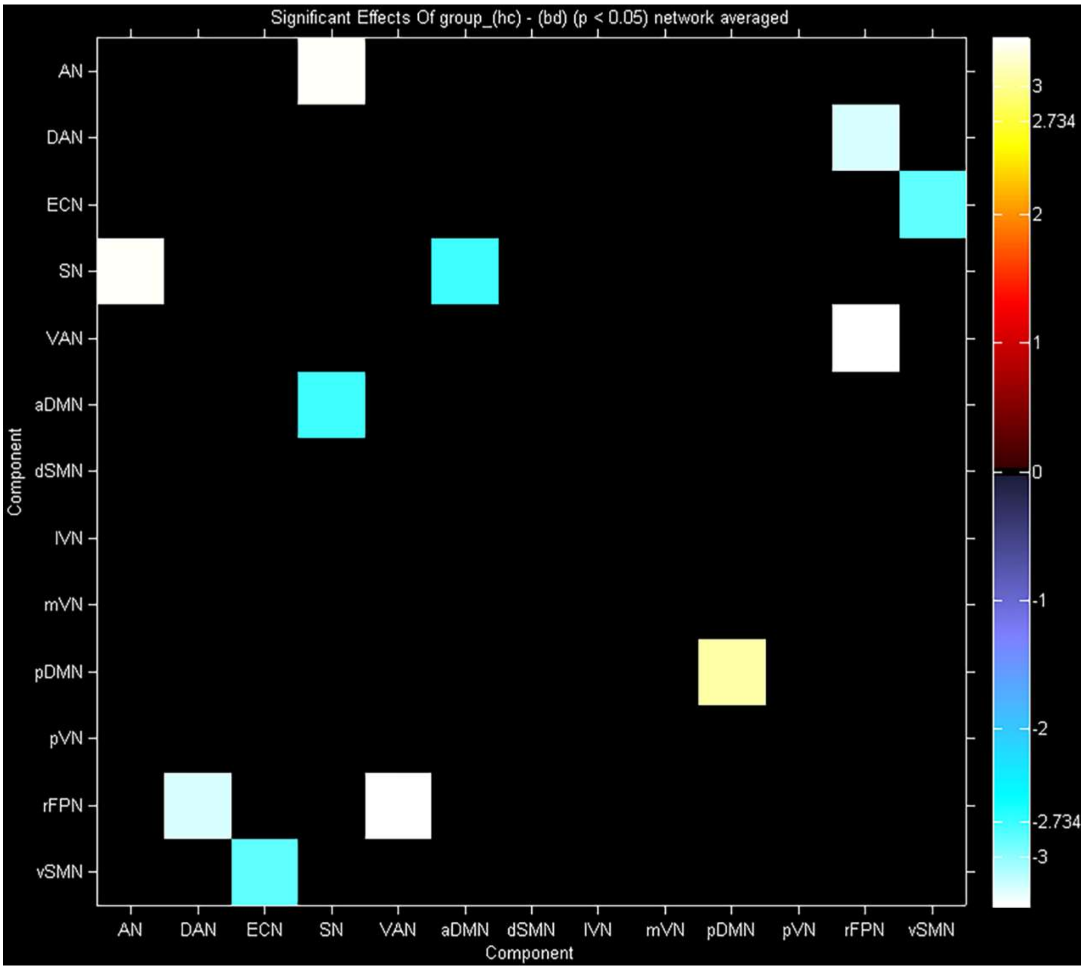

Figure S9. The effect of experimental group and control group was significant (between brain networks).

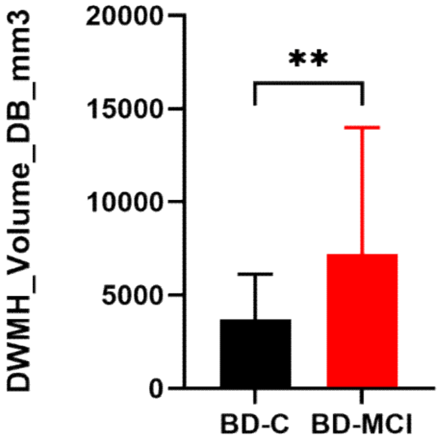

Figure S10. The analysis of WMHs revealed that the BD-MCI group exhibited a significantly greater volume of DWMH.

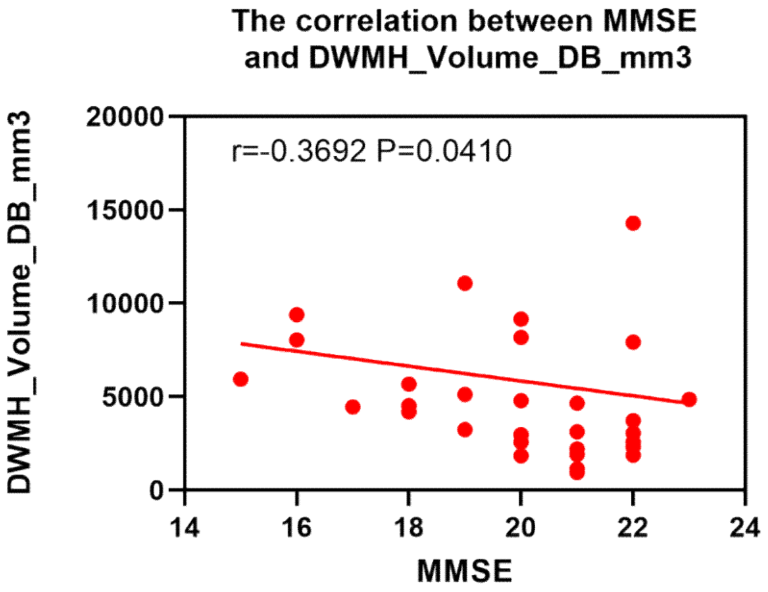

Figure S11. The volume of DWMH was negatively correlated with MMSE score.
